# Supplementary material for: Are carbon emissions trading and green financial instruments synergistic? -Comprehensive quantitative research based on content analysis
Source: PLoS One. 2024 Mar 7;19(3):e0298601. doi: 10.1371/journal.pone.0298601 (PMC10919601; doi:10.1371/journal.pone.0298601)
Supplement: S1 Data — (ZIP) [file pone.0298601.s001.zip › Data/地级市Z_F工作报告.zip!/─┌├╔╣┼/═¿┴╔╩╨/└┤╫╘╣1⁄2╓┌║┼í╛┬φ┐╦╩2╛▌═°í┐_2015.docx]

一、2014年工作回顾

过去的一年，是我市发展进程中具有里程碑意义的一年。面对复杂严峻的经济形势和艰巨繁重的改革发展任务，市政府在自治区党委、政府和市委的正确领导下，团结带领全市各族人民，深入贯彻习近平总书记系列重要讲话和考察内蒙古重要讲话精神，全面落实自治区“8337”发展思路和市委各项决策部署，主动适应经济发展新常态，审时度势，攻坚克难，经济社会发展取得了新成效。

（一）坚持稳中求进工作总基调，经济实现持续稳定增长。积极应对经济下行压力，认真落实市委、市政府确定的43项重点工作行动计划，经济实现稳中有进。“稳”主要体现在：主要经济指标平稳增长，地区生产总值完成1886.8亿元，增长8.6%，总量排全区第四位、增速排全区第二位；限额以上固定资产投资完成1866.4亿元，增长18.3%，总量和增速均排全区第三位；公共财政预算收入完成113.1亿元，增长9.1%，公共财政预算支出318.2亿元，增长6.6%，其中，民生支出190.2亿元，占总支出的60%；社会消费品零售总额完成419.4亿元，增长11.2%；就业形势稳定，新增就业超额完成年度计划。“进”主要体现在：结构调整迈出新步伐，第三产业占经济总量的比重达到29.3%，提高5.5个百分点；落实各项扶持措施，规模以上工业增速逐步企稳回升，全年增长10.9%；县域经济发展实力和活力明显增强，旗县市区工业园区产值均超百亿元，霍林郭勒市工业园区被确定为国家级新型工业化产业示范基地。8个旗县市区公共财政预算收入全区排名均有提升。

（二）推进“三大产业集群”建设，转方式、调结构取得积极进展。以“三大产业集群”建设为抓手，优化空间布局，调整产业结构，促进发展方式转变。工业转型升级加快。强化要素支撑和创新驱动，火电、风电、光伏互补的霍林河循环经济示范项目获联合国气候大会“今日变革进步奖”，探索出了高载能产业实现绿色低碳发展的新路子。实施工业延伸升级项目352个，福耀汽车玻璃、欣意高端电缆等一批重点项目建成投产。铝新材料新增产品137个，累计达到260个品种，原铝转化率达到80%。玉米生物科技新增产品55个，累计达到119个品种，玉米淀粉转化率达到53%。现代煤化工、新能源、绿色农畜产品加工、现代蒙医药等产业快速成长。农牧业现代化取得重要成果。新建节水高产高效粮食功能区170万亩，累计建成651万亩。压减机电井8305眼，累计压井2.2万眼，年农业节水4亿立方米。粮食产量位居全区首位，达到132.6亿斤，连续7年稳定在百亿斤以上。牧业年度牲畜存栏首次突破2000万头只，达到2006.9万头只。牛存栏达到275万头，增长8.3%。农牧业标准化、品牌化建设力度加大，在北京、呼和浩特成功举办绿博会，提升了我市绿色农畜产品的知名度和市场占有率。服务业增长势头良好。旅游、物流、电子商务、金融等服务业加快发展，成功举办中国蒙餐烹饪大赛、全区第六届乌兰牧骑艺术节、哲里木赛马节等节庆活动，我市被命名为“中国蒙餐之都”。旅游业收入增长25%，物流业增加值增长12%，社会融资总量新增273亿元，民航机场客运量达到70.9万人次，增长23.8%。城乡统筹步伐加快。落实主城区城市建设管理、产业发展及民生改善一揽子行动计划，开工建设了新世纪大桥、孝庄河南北路等桥梁道路工程，实施了12个民族文化专题博物馆和科技馆、图书馆、老年人活动中心等社会事业工程。辽河公园、胜利河和孝庄河休闲景观带基本建成，完成了主城区外环线、街角游园等重点绿化工程。通过不懈努力，成功创建国家园林城市。统筹推进县城、特色镇、美丽乡村建设，舍伯吐城乡一体化示范镇建设成效明显。

（三）全面深化改革开放，发展动力和活力持续增强。聚焦重点、精准发力，各项改革举措落地见效。加大简政放权力度，市本级行政审批事项减少91项，精简幅度达到31%，行政审批时限压缩54%。实施工商登记便利化改革，全市新增市场主体3.8万户，总量达到16万户。落实大用户直供电量30.3亿度，占蒙东地区交易量的90%以上，北部区域微型电网试点列入国家振兴东北发展战略。加强通辽科技大市场等创新平台建设，有效促进了关键技术攻关和科技成果转化。发展适度规模经营，农村牧区多种形式流转土地715万亩。助农金融服务网点实现行政嘎查村全覆盖。启动与俄蒙对接合作，外贸进出口总额实现翻番。围绕“三大产业集群”，大力开展招商引资，产业层次和对外开放水平进一步提高。

（四）加强基础设施建设和生态环境保护，发展保障能力不断提升。开工建设二级以上公路208公里，新建通村公路1247公里，通畅率达到64%，提高10个百分点。霍林郭勒市珠斯花至锡林郭勒盟珠恩嘎达布其口岸铁路全线通车。霍林郭勒民用机场主体工程完成过半。辽宁昌图至通辽天然气管道全线贯通并具备供气条件。启动建设科尔沁沙地“双千万亩”综合治理和生物多样性示范区工程，完成植树造林193万亩，草原建设679万亩，退耕还林还草155万亩。城郊百万亩森林建设任务全部完成，罕山自然保护区晋升为国家级自然保护区。开展环保整治“百日行动”，对存在环境隐患和重大问题的园区、企业及项目进行挂牌督办，严格惩处环境违法行为。推进大气污染防治和重点流域、重点区域环境综合治理，节能减排和降碳完成自治区下达的目标任务。

（五）加大保障和改善民生力度，群众生活水平逐步提高。城镇和农村牧区常住居民人均可支配收入分别达到23377元和9932元，分别增长9.5%和11.3%。全面完成农村牧区700个嘎查村“十个全覆盖”工程任务，全区现场观摩会在我市召开。新增城镇就业1.8万人，实现零就业家庭动态清零。农村牧区劳动力转移就业48.3万人。实施规划、项目、干部“三到村、三到户”精准扶贫和“金融扶贫富民”工程，1.8万户、7万贫困人口实现稳定脱贫。改造城市棚户区3万户、农村牧区危土房3.5万户，新建公共租赁住房1100套。开工建设居民之家30个，建成村牧民之家108个。落实“一吨暖心煤”和贫困大学生救助政策，临时救助、医疗救助及残疾人“五项工程”惠及弱势群众43.7万人次。新农合制度不断完善，引入商业保险参与经办服务，提高大病最高支付限额，城乡低保社会保障标准和补助水平稳步提高。

（六）发展各项社会事业，社会保持和谐稳定。进一步完善教育规划布局，深化教育领域综合改革，各级各类教育持续均衡发展。在全区率先实现了每个苏木镇、中心嘎查村都有一所公办幼儿园的目标。强化市级医疗卫生机构与北京市部分医院合作，带动医疗技术水平不断提高。市健康体检中心投入使用，旗县医院整体达到二甲水平。各项人口计划生育目标圆满完成。完成广播电视户户通工程4万户，建设地面数字电视发射基站11个，使20万农牧民观看到通辽市和本地区电视节目。建成一批文化活动室和文体广场，开展系列文化惠民活动和“三大球”联赛，城乡居民文体活动日益活跃。通辽市潮尔、库伦旗布鲁被评为中国第四批非物质文化遗产，国家级非物质文化遗产达到11项、传承人达到9名。认真贯彻落实中央民族工作会议精神，发展民族团结进步事业，平等团结互助和谐的社会主义民族关系进一步巩固。新闻出版、人民防空、外事、侨务、宗教、气象、地震、档案、工会、共青团、妇女儿童、老龄、慈善、关心下一代等事业取得新成效。

深化平安通辽建设。推行社区“网格化”管理和嘎查村“532”工作法，基层法律服务实现全覆盖。完善立体化社会治安防控体系，严厉打击违法犯罪活动，社会大局和谐稳定。推进信访改革，依法规范信访秩序，妥善化解了一批信访积案。实施食品药品监管体制改革，加大专项检查力度，保障食品药品安全。狠抓安全生产监管和事故隐患排查整治，安全生产形势稳定。积极支持国防和军队现代化建设，深入开展国防教育和双拥共建，国防动员和后备力量建设取得新进展。

各位代表，一年来，我们认真执行市人大及其常委会决定，自觉接受政协民主监督，广泛听取各民主党派、工商联、无党派人士和市民代表意见，及时办结人大代表议案、建议和政协委员提案。深入开展党的群众路线教育实践活动，严格落实中央八项规定、自治区28项配套规定和市委有关规定，坚决反对“四风”，着力打通服务群众“最后一公里”，集中解决了一批关系群众切身利益的突出问题。全市收缴封停超配越野车324辆、拍卖超标奥迪牌公务用车160辆，拍卖资金全部用于购置公交车和校车。全市机关单位“三公”经费支出大幅压减，节约资金主要用于改善民生。认真执行“三重一大”制度，进一步完善政府议事规则和决策程序。推进政府系统廉政建设，开展正风肃纪活动，加大审计监督、行政监察和政务公开力度，严肃查处违纪违法案件，以作风建设的新成效汇聚推动改革发展的新能量。

各位代表，过去的一年令人难忘。全市各族干部群众在困难和压力面前，勇于担当，奋力拼搏，创造了“扎鲁特现象”、“霍林河模式”、“科尔沁速度”、“舍伯吐精神”，为我们积累了宝贵经验。这些成绩的取得，是自治区党委、政府和市委正确领导的结果，是社会各界关心、支持、帮助的结果，是全市各族人民守望相助、团结奋斗的结果。在此，我代表市人民政府，向全市各族干部群众，向所有关心支持通辽建设与发展的同志们、朋友们，致以崇高的敬意和衷心的感谢！

在看到成绩的同时，我们也应该认识到，我市经济社会发展中还存在着一些不容忽视和亟待解决的困难和问题。主要是：稳定经济增长压力较大，受产能过剩和市场需求低迷影响，我市主要工业产品价格下降、企业资金链紧张、房地产市场不够活跃，部分企业生产经营还比较困难。结构调整任务依然艰巨，农牧业基础仍然薄弱，资源利用方式还较为粗放，创新能力不强，现代服务业发展相对滞后，城镇化水平还不高，城乡区域发展不平衡。民生和社会建设力度还需加大，全体居民收入与自治区平均水平还有一定差距，部分群众生活仍比较困难。城乡公共服务设施欠账较多，教育、医疗卫生、文化体育等基本公共服务供给不足。政府自身建设还需要进一步加强，政府职能转变和工作作风还不能很好地适应经济发展新常态的要求，少数部门行政行为不规范、办事效率低，一些干部改革创新和敢于担当意识不强。对于这些问题，我们要以时不我待的紧迫感和责任感认真加以解决。

二、2015年主要工作任务

2015年是全面深化改革的关键之年，是全面推进依法治市的开局之年，也是全面完成“十二五”规划的收官之年，做好今年工作意义重大。2015年政府工作的总体要求是：全面贯彻落实党的十八大和十八届三中、四中全会及中央经济工作会议精神，深入贯彻习近平总书记系列重要讲话和考察内蒙古重要讲话精神，全面落实自治区党委九届十三次全委会议暨全区经济工作会议、自治区十二届人大三次会议和市委四届十次全委会议暨全市经济工作会议精神，坚持稳中求进工作总基调，主动适应经济发展新常态，牢牢把握发展主动权，坚持“一个中心”，加快“四个转向”，建设“三大产业集群”，做好“八个方面工作”，统筹推进转方式、调结构、促改革、惠民生，促进经济社会转型发展、科学发展、率先发展。

坚持“一个中心”，就是围绕全面建成小康社会的目标，坚定不移把发展作为第一要务，以提高经济发展质量和效益为中心，稳中求进，跨越发展。加快“四个转向”，就是加快经济增长速度从高速增长向中高速增长转向；经济发展方式从规模速度型粗放增长向质量效率型集约增长转向；经济结构从增量扩能为主向调整存量、做优增量并举转向；经济发展动力从传统增长点向新的增长点转向。建设“三大产业集群”，就是加快建设北部铝新材料、现代煤化工产业集群，中部自主创新和现代商贸物流中高端产业集群，南部融入东北一体化和绿色产业集群。做好“八个方面工作”，就是着力做好保持经济持续健康发展、优化经济结构、发展现代农牧业、推进新型城镇化、深化改革开放、保障改善民生、发展社会事业、强化政府自身建设等八个方面工作。

今年，全市经济社会发展的主要预期目标是：地区生产总值增长8%，公共财政预算收入增长6%，固定资产投资增长13%，社会消费品零售总额增长11%，城镇和农村牧区常住居民人均可支配收入分别增长10%和11%以上，完成自治区下达的节能减排和降碳目标任务。

这些预期目标的确定，充分考虑了经济发展新常态的内在要求和趋势性变化，统筹考虑了我市经济发展的阶段性特征和外部环境影响，并与编制“十三五”规划和全面建成小康社会目标相衔接，是实事求是、积极稳妥的，也是需要艰苦努力才能实现的。当前，我国经济发展进入新常态，这是以习近平同志为总书记的党中央做出的重大战略判断，是当前和今后一个时期我国经济发展的大逻辑，我们必须把思想行动统一到中央、自治区和市委关于新常态的分析判断和部署要求上来，牢牢把握发展主动权。一是正确认识新常态。深刻领会新常态的思想内涵和实践要求，充分认识新常态下速度变化、结构调整、动力转换的特点，做到观念上适应、认识上到位，保持经济增长在合理区间，切实加快转方式调结构，推动产业向中高端水平发展，做到调速不减势、量增质更优。二是主动适应新常态。准确把握新常态下经济发展的趋势变化，做到方法上对路、工作上得力，从过去习惯于铺摊子、上项目转到主要依靠改革创新上来，用改革的思路和措施，加快转变经济发展方式，调整优化经济结构，发现和培育新的增长点。三是积极引领新常态。按照习近平总书记新常态“八个更加注重”的要求，准确把握发展大势，切实提高预见性、把握规律性、增强主动性，抢抓新机遇，应对新挑战，与时俱进地开创我市经济社会发展新局面。

（一）保持经济持续健康发展，实现稳中求进

稳增长的同时，实现跨越发展，需要我们抓住国家产业调整和扩大内需的机遇，更好地发挥投资的关键作用和消费的基础作用。

保持投资稳定增长。围绕产业升级、基础设施、社会事业、民生工程、生态建设和城镇建设等投资领域，开工建设一批重点项目。工业方面，新开工和续建500万元以上工业重点项目280个，完成工业固定资产投资600亿元以上。重点抓好霍林郭勒市锦联铝板带箔、创源高强高韧铝合金、梅亿新能源汽车，科尔沁区梅花生物小品种氨基酸，科左中旗科尔沁牛业肉牛产业园，库伦旗荞泰生物荞麦深加工，扎鲁特旗龙源绿美褐煤综合利用，科左后旗康臣药业口服液，开鲁县华曙制药、黄河龙黄原胶，奈曼旗和谊镍铬复合材料，开发区金煤化工技改等项目。基础设施方面，统筹推进微电网、电源点和外送电力通道建设，开展区域微型电网试点，加快推进通辽至河南特高压电力外送通道项目前期工作，配套建设一批风电、光伏、火电互补的电源点项目。开工建设通辽至京沈客专连接线、通辽至鲁北高速公路和主城区北外环连接线工程，完成霍林郭勒市至兴安盟阿力得尔一级公路、科左中旗舍伯吐至大兴（通赤界）二级公路、霍林郭勒市至锡林郭勒盟巴彦胡硕二级公路改造续建项目，霍林郭勒民用机场建成运营。开工建设扎鲁特旗毛都水库，推进“引绰济辽”等大型跨流域调水工程和库伦旗嘎海山水库前期工作。民生和社会事业方面，开工建设全民健身中心、蒙医中心医院、市医院门诊综合楼、市儿童医院、新城区实验小学等项目，完成“四校合一”建设任务。建成12个民族文化专题博物馆并力争投入使用，完成那达慕文化园、科技馆、图书馆、城市规划馆、老年人活动中心主体工程建设。

培育新的消费热点。更加注重市场和消费心理分析，适应消费模式转型升级的新趋势，着力培育新兴业态。促进文化旅游深度融合，启动建设“科尔沁500公里文化旅游风景大道”，建设大青沟、珠日河、可汗山等6个自驾车营地和扎鲁特山地草原慢行系统，打造自驾游精品线路。加快建设库伦旗银沙湾旅游区、科左后旗阿古拉湿地公园、科尔沁区莫力庙环湖自行车赛道、主城区辽河公园环湖步道等项目。推动孝庄园、三大寺、孝庄河文化休闲商业街5A级景区创建工作。依托深厚的科尔沁历史文化底蕴，打造一批科尔沁文化旅游精品。结合智慧旅游系统建设，举办系列文化旅游推介活动。大力发展现代物流业，以建设东北区域物流中心为目标，对主城区物流园实施升级改造，建立覆盖城乡的现代物流体系。重点推进“一港四园四中心”建设，“一港”即完成通辽内陆港四期扩能改造，进一步扩大东北货运快车货物吞吐量。“四园”即加快开发区综合物流园、空港物流园、科尔沁综合物流园和木里图工业物流园建设。“四中心”即促进内陆港保税仓库升级为保税物流中心，加快建设通辽国际快件监管中心、蒙东煤炭交易中心和区域性日用消费品配送中心。同时，加快推进各旗县市区物流园区和物流项目建设。积极培育新兴服务业态，扶持发展电子商务、健康养老、体育健身等现代服务业。建设通辽电子商务产业园区和400个嘎查村电商服务示范点，全市电子商务交易额力争突破10亿元、电商企业数量达到200家以上。引导支持房地产市场健康发展，盘活住宅存量，建设绿色低碳住宅，带动相关产业发展。

推进融资方式多元化。发挥金融机构主渠道作用，提高金融服务水平。推进政银企互动，在企业和银行间搭建平台，加大政府性担保资金投入，健全中小企业信用担保体系，发挥信用担保、贷款贴息等政策工具的作用。大力推广政府和社会资本合作模式，公布面向社会投资领域的合作项目名录，引导社会资本投向基础设施等领域。支持有条件的中小企业通过创业板和全国中小企业转让系统进行上市融资和股权融资。鼓励民间资本发起设立投资基金，规范民间融资。调动全社会融资积极性，把融资作为招商引资的重点，吸引更多的社会资本参与我市重点项目建设。

着力稳定经济运行。以稳定工业运行和企业生产经营为重点，加强经济运行调度，帮助企业减负增效，促进新投产项目达产达效，稳定经济增长基础。用好电力综合扶持政策，有效降低企业用电成本。公布涉企收费目录清单，切实做到清单之外无收费。支持企业加强上下游产业协作，提升产业间的依存度和抗风险能力。

（二）优化经济结构，加快转变经济发展方式

推进产业结构转型升级。把延长资源型产业链条作为我市调整优化产业结构的重要内容，加快产业延伸升级，提高资源综合利用率，做好资源转化和精深加工的文章，构建多元发展、多极支撑的现代产业体系。加快传统产业新型化。鼓励煤企、电企和铝精深加工企业开展纵向合作，推广高载能产业低碳循环发展模式，抓好蒙东能源循环经济示范项目，形成产业链成本竞争优势。加快玉米生物科技、绿色农畜产品加工等传统产业延长产业链条，提高产品附加值，实现转型升级。推进新兴产业规模化。重点推动扎鲁特旗康乃尔30万吨乙二醇等项目投产，增加现代煤化工产业链，打造现代煤化工生产示范基地。加快蒙药股份与科尔沁药业重组上市，促进库伦蒙药等企业做大做强。加快建设伯恩露笑蓝宝石晶体、欣意稀土高铁铝合金电缆等项目，做大新兴产业规模。推动支柱产业多元化。扶持现代装备制造、新能源、新材料等产业发展，开发新产品、发展新业态，打造新的支柱产业。

坚定不移推进“三大产业集群”发展。加快打造北部铝新材料、现代煤化工产业集群，以霍林郭勒市、扎鲁特旗为重点，依托已经建成的工业园区和重大项目，切实抓好一批铝精深加工项目，延伸产业链条，加快有色金属基地建设步伐。加快打造中部自主创新和现代商贸物流中高端产业集群，以科尔沁区、开发区、开鲁县为重点，依托自主创新产业园区和重点工业园区，抓好现代蒙医药、食品加工等重点项目，集聚科技、金融、信息等高端要素，建设区域性物流枢纽城市和科技创新城市。加快打造南部融入东北一体化和绿色产业集群，以科左中旗、科左后旗、库伦旗、奈曼旗为重点，依托毗邻东北的区位优势，以承接东北三省产业转移、建设绿色产业示范带为突破口，加快绿色农畜产品加工园区建设步伐。大力发展非资源产业，积极引进机械加工、精密铸造、重型机械组装等项目，做大特色装备制造产业，建设南部四旗绿色产业和融入东北的开放经济带。

加快创新能力建设。创新驱动是转变发展方式，提高经济发展质量的重要依托和动力源泉，必须摆在重要位置加以推进。强化科技创新平台载体的创新引领作用，抓好通辽科技大市场、内蒙古蒙医药工程技术研究院、蒙东铝新材料工程技术研究院、生物高科技创新园等建设，推进产学研协同发展。抓好关键技术创新，围绕现代煤化工、玉米生物、肉牛、硅砂等重点产业，开展关键技术攻关和大数据技术的应用，打造一批掌握核心技术、引领行业发展的创新型企业，培育一批具有自主知识产权的中小型企业，真正让企业成为创新主体，使创新成果转化为现实生产力。优化科技创新环境，深化科技体制改革，落实与内蒙古民族大学全面战略合作协议，深化与中科院、清华大学等知名院校和北京西城区、西安高新区等先进地区的科技合作与交流，加大知识产权的保护、利用和开发。加快实施人才强市工程，建立人才信息化服务网络和平台，引进培养中高端人才。培养和引进蒙医药人才，支持名老蒙医药专家带徒授业。利用院士工作站、博士后工作站和创新工作室，创办区域性“人才改革试验区”，发挥人才对经济社会发展的引领作用。

（三）大力发展现代农牧业，推动农牧业大市向农牧业强市转变

认真贯彻落实中央1号文件精神，把“三农三牧”作为全市工作的重中之重，按照“稳粮增收、提质增效、创新驱动”的总要求，转变农牧业发展方式，推进农牧业现代化进程。

加快转变农牧业发展方式。应对土地、环境和水资源的多重约束，转变拼资源、拼农资投入、拼生态环境的粗放经营发展方式，加快节水、退耕、轮牧、还绿步伐，走产出高效、产品安全、资源节约、环境友好的现代农牧业发展道路。落实11个农村牧区重点工作行动计划，全面完成市第四次党代会提出的“8511521113”农牧业现代化工程建设任务。新建节水高产高效粮食功能区200万亩、达到851万亩。压减机电井8350眼，累计达到3万眼，实现农业节水5.4亿立方米。在科左中旗舍伯吐项目区抓好水利部定点节水农业改革试点。发展以肉牛为重点的现代畜牧业。建设存栏1000头的科尔沁肉牛核心育种场，实施母牛扩繁“万千百十”示范工程。全市牛存栏新增26万头、总量突破300万头。调整种养结构、产品结构和区域结构，提高畜牧业在大农业中的比重，大力发展农区畜牧业，推广规模化、专业化、工厂化等新型养殖模式，把粮食和秸秆转化成肉蛋奶，提高农畜产品附加值。创新发展生态草原畜牧业，落实草畜平衡和禁牧轮牧措施，建设饲草料基地500万亩，牲畜头数稳定在2000万头只以上。

推进农牧业标准化和品牌化建设。围绕建设绿色农畜产品生产加工输出基地，大力推进农牧业标准化生产。进一步修订完善农牧业生产标准，新建玉米、荞麦、肉牛、肉羊等8个优势特色产业标准化示范基地。强化农资市场管理，做好动物疫病防控和农作物病虫害防治，提升农畜产品质量安全监管和检测能力。抓好肉牛质量安全追溯系统建设，年内实现5万头育肥牛的质量安全可追溯。更加注重品牌化发展，加强生态原产地产品保护和申报工作，培育更多的农畜产品品牌。

创新农牧业生产经营方式。坚持农牧民家庭经营主体地位，在尊重农牧民意愿的前提下，引导农村牧区土地经营权规范有序流转，提高农牧民组织化程度。发展适度规模经营的家庭农牧场，培育壮大龙头企业和合作经济组织，建立完善与农牧民利益联结机制，大力发展产业化经营。加快发展“博览”经济，创新流通方式和业态，大力推进电子商务与实体流通相结合，推动优质绿色农畜产品走出通辽，走向全国。

（四）积极稳妥推进城镇化，促进城乡统筹发展

落实市委、市政府《关于推进新型城镇化的实施意见》和主城区2015年城市规划建设管理行动计划，加快构建绿色、人文、现代的区域性中心城市和实力县城、风情小镇、美丽乡村相互呼应的新型城镇化体系。

创建科尔沁文化旅游名城，加快区域中心城市建设。把打造科尔沁文化旅游名城作为通辽城市转型发展的重要突破口，将科尔沁文化的精髓融入到城市规划建设中，提升城市文化品位，延续好历史文脉，让城市留得住记忆，让百姓记得住乡愁。加强城镇规划。完成《通辽市城市总体规划（2014—2030年）》修编工作，加大主城区控制性详规和专项规划编制力度，加强各旗县市和重点镇、村规划工作，提高规划覆盖率，让规划引领城市未来。完善城市功能。主城区实施“两环、两桥、六出口”等城市道路桥梁畅通工程和“四街路”改造提升工程。也就是，打通以新世纪大桥为核心的城区内环路，建设以主城区周边高速和一级路为框架的外环路；确保新世纪大桥主体完工，启动建设红光大桥；完成霍林河大街东段及东出口拓宽改造、叫来河大街西段公铁立交桥、机场路及通赤高速出入口环境改造工程，推动建国北路、胜利北路拓宽改造项目开工建设。打通8条断头路、改造提升既有道路20条，建设20公里城市慢行系统。对霍林河大街、建国路、胜利路、创业大道等四条街路进行综合改造。巩固发展园林城市建设成果，实施辽河公园扩建、孝庄河、胜利河沿岸园林景观、明仁公园建设等项目。推动万达广场、万力城、五洲国际商贸城等城市综合体尽快建成运营。对主城区小街小巷进行综合整治，实现小街小巷硬化亮化全覆盖。加大老旧住宅小区改造和物业服务管理力度，保障住宅小区供热、供电、给排水、路面硬化等基本公共服务，切实提高住宅小区物业服务管理水平。建设实力县城和风情小镇，旗县市实施“一街、一路、一镇、一社区”改造工程。结合实施农村牧区“十个全覆盖”工程，推进美丽乡村建设，逐步改善乡村面貌。加强城市管理。一座城市，三分建、七分管。管理好城市是广大市民对政府的热切期盼。今年，我们要进一步理顺城市管理体制，强化监管，推动科尔沁区和开发区同城同步协调发展；加快“智慧城市”建设，提升城市管理水平，实现城市管理的信息化、精细化；推进城市管理综合执法，开展城市违法违规建筑专项整治行动；积极开展爱国卫生运动，实施城乡清洁行动计划，认真落实“门前三包”，调动和激发群众参与城乡管理的积极性，像爱护家园一样，呵护我们的城市。

发展壮大县域经济。按照主体功能区定位要求，结合特色园区建设和城镇扩容提质，促进产城融合，提高旗县市区工业园区承载能力，促进产业培育、园区建设、城镇功能协调发展。建立市政府和各旗县市区政府工作联动机制，坚持问题导向，抓好“扩权强县”改革试点，共同破解发展难题，提高县域经济综合实力。

加强生态文明建设。重视生态工程建设，最大限度增加生态资产。执行最严格的耕地保护制度和资源节约集约利用制度，坚守耕地红线。加快创建国家森林城市步伐，实施科尔沁沙地“双千万亩”综合治理工程，抓好生物多样性保护示范区、退耕还林还草、生态脆弱区移民搬迁、矿山地质环境治理等重点生态工程建设，年内完成科尔沁沙地综合治理355万亩、退耕还林还草100万亩。保护利用好扎鲁特山地草原。建设科尔沁生态科技示范园，促进生态科技成果推广应用。强化生态文明制度建设，最大限度减少环境负债。全面贯彻新修订的《环境保护法》，强化大气、水、土壤和重金属污染综合防治，加强减排治污重点工程建设，下大力气解决损害群众健康的环境污染问题。加大环境综合整治和执法力度，对环境违法行为“零容忍”。严格治理“滥开垦、滥放牧、滥樵采”。抓好农牧业清洁生产、工业循环利用示范项目和示范基地建设，大力发展循环经济。实行最严格的水资源管理制度，建立用水总量控制和用水效率体系，强化地下水节约保护。抓好编制自然资源资产负债表、领导干部自然资源资产离任审计、生态环境损害责任终身追究制度三项重点任务，认真落实资源有偿使用和生态补偿制度，推进生态文明建设制度化和法治化。

（五）全面深化改革开放，增强发展新动力

正确处理政府与市场的关系，以深化改革和扩大开放为突破口，最大限度激发市场和社会活力。

深化重点领域和关键环节改革。一是深化行政审批制度改革。推进简政放权，把政府该放的坚决放到位，把政府该管的坚决管好。对保留的行政审批事项，一律纳入政务服务中心集中办理，不搞“体外循环”。深化商事制度改革，加快推行企业注册登记“三证合一”。全面推行网上审批，大幅压缩审批时限，提高行政效率。二是加快电力自营区建设。进一步拓展大用户直供电范围和规模，积极推进北部区域微型电网试点工作。三是深化国有企业改革。推动霍煤集团建立现代企业制度，加快所属企业混合所有制改造。四是深化农村牧区综合改革。开展农村牧区金融改革试点，巩固扩大集体林权制度改革成果，积极开展林权抵押贷款、森林保险等相关配套改革。全面启动农村牧区小型农田水利工程产权改革，完成农村牧区土地承包经营权确权登记颁证试点和半农半牧区草原划定及草牧场确权承包工作。加快北部牧区现代化和现代农牧业综合改革发展示范区建设。五是深化财税管理体制改革。认真贯彻执行新《预算法》，改进预算管理制度，建立跨年度预算平衡机制，将政府收支全部纳入预算。优化财政支出结构，保障重点支出。对政府债务实行规模控制和分类管理，规范政府举债行为。坚持依法理财，确保财政资金阳光运行。

以扩大开放增添发展活力。加快打造内陆沿边开放新高地。抓住国家实施“一带一路”战略、自治区建设向北开放桥头堡和沿边经济带新机遇，以建设“三大产业集群”、推动产业转型升级为目标，实施更加积极的开放战略，主动融入国家、自治区开放发展战略全局，面向国内国际两个市场，以开放促转型、促发展，构建全方位、多层次、宽领域的对外开放格局。深化与俄蒙开放合作。落实市委、市政府《关于进一步加强同俄罗斯和蒙古国合作实施意见》，打造草原丝绸之路重要节点城市，全方位开展与俄罗斯和蒙古国合作。协调建设口岸服务功能区和保税库，支持企业在俄蒙设立营销中心或加工基地，发展进出口贸易和加工贸易。加快推进与俄蒙基础设施的互联互通，扩大商贸往来和人文交流，建立长期合作机制。全方位推进对外开放。加强与沿海发达地区、东北经济区、环渤海、东北亚等海内外大市场的对接协作，有针对性地开展招商活动，既抓产业延伸升级的实体项目引进，也抓资本、科技、人才等要素引进，在深化合作中实现互利共赢。

促进非公经济健康发展。非公经济是扩大就业的主力军，要毫不动摇地巩固和发展非公经济。着力优化发展环境，落实各项扶持政策，加快公共服务体系建设，完善非公经济阳光服务中心功能，建立政策信息综合服务平台，为非公经济发展提供全程服务。加大行业异地商会组建力度，发挥行业协会、商会等社会中介组织和民间团体的行业自律和服务功能，培养一批具有现代经营管理水平的企业家队伍。

（六）回应群众期盼，切实保障和改善民生

增加城乡居民收入。落实国家和自治区深化收入分配制度改革相关政策，适时调整机关事业单位基本工资标准。完善企业工资集体协商和工资正常增长机制。认真落实惠农惠牧政策，多渠道促进农牧民增收。

健全社会保障体系。完善新农合制度，提高新农合补助资金和住院报销比例。推进重大疾病商业保险工作，提高城镇职工医疗保险统筹基金年度最高支付限额。做好进城农民工养老保险转移接续工作，调增城镇居民基础养老金标准。提高城乡低收入群体保障标准和补助水平，完善临时救助制度，给予困难家庭及个人更加有效的应急救助。重视老龄问题，加强养老基础设施和服务体系建设，提高老年人生活质量。

推进重点民生工程建设。实施农村牧区“十个全覆盖”工程，整合资金40.5亿元，完成764个行政嘎查村“十个全覆盖”工程，并在此基础上按照“7个100%、20项全覆盖”标准，建设100个新农村新牧区示范嘎查村。加强后续管理和制度建设，确保公共服务设施长期发挥使用效益。实施就业创业工程，培育通辽人才大市场，实施“三大产业集群”带动就业30万人三年行动计划，新增就业1.7万人，农牧民转移就业46万人，开展就业创业培训3万人。实施精准扶贫工程，稳步推进“三到村、三到户”精准扶贫和“金融扶贫富民”工程，完成年度扶贫任务。实施百姓安居和居民之家建设工程，改造农村牧区危土房3.5万户，基本完成全市农村牧区危土房改造任务。改造城市棚户区2万户。全市新建社区居民之家39个，主城区实现居民之家全覆盖。实施安全饮水工程，新建农村牧区安全饮水工程226处。完成主城区第五水厂改扩建工程，日供水能力由3.5万吨提高到5万吨。实施食品药品安全市建设工程，建立食品生产质量安全可追溯体系，启动建设餐厨废弃物、废弃油脂处理厂，各旗县市区食品加工园全部投入使用。加大食品药品安全专项整治力度，全面规范食品药品市场秩序，到2017年基本建成食品药品安全市。实施基本医疗保健和医疗保险统筹工程，优化医疗卫生资源配置，实现嘎查村标准化卫生室全覆盖。确保上半年完成医疗保险市级统筹工作。实施文化体育惠民工程，加大文化下基层工作力度，公共文化服务设施全部免费对外开放。新建一批公共健身场所。部分机关、企事业单位健身场馆免费向社会开放，校园体育设施全年向学生开放。实施公交便民工程，加快主城区公交城乡一体化进程，对公交线路和公交车实行统一的管理体制。更新一批新型公交车，逐步实现主城区公交车智能化管理。对减免费乘坐公交车的群体，落实国家政策，兑现政府承诺。实施便民公厕工程，主城区现有公厕全部免费开放，通过新建、改造、购买、协调临街单位开放厕所等方式，实现主城区公厕全覆盖。

（七）大力发展社会事业，加强社会治理创新

加快各项社会事业发展。坚持优先发展教育事业，推进基础教育城乡一体化，加快义务教育均衡发展。规范办学行为，解决好“择校热”、“大班额”等问题。促进民族教育内涵发展，探索建立职教发展新模式。深化旗县级公立医院综合改革，推进医疗卫生事业发展。坚持计划生育基本国策，提高出生人口素质。加快推进公共文化服务体系标准化、均等化建设，促进文化繁荣发展。实施足球改革发展行动计划，以足球等“三大球”联赛带动大众健身体育发展。进一步加强妇女儿童、外事侨务、残疾人、地震、人防和气象等工作。深入开展社会主义核心价值观、“中国梦·尽责圆梦”系列宣传教育和主题实践活动，丰富群众性精神文明创建内涵。推动传统媒体和新媒体融合发展，做好网络信息管理和舆情引导工作，利用微博、微信等现代媒体，宣传通辽新形象，凝聚发展正能量。

做好民族宗教工作。全面贯彻落实党的民族宗教政策，积极开展民族团结进步创建活动，促进各民族共同团结进步、共同繁荣发展。加强蒙古语文法制化、规范化、标准化和信息化建设。发挥宗教界人士和信教群众在促进社会和谐稳定中的积极作用。

推进平安通辽建设。全面实施依法治市，开展以宪法为核心的全民法治宣传教育，深入推进“六五”普法，切实增强全民法治观念和法治素养。坚持依法规范信访工作秩序，健全依法维权和化解纠纷机制，保障群众合理合法诉求通过法治渠道得到解决。建立警力下沉机制，打造警务共同体，完善立体化社会治安防控体系。严厉打击各类违法犯罪活动，确保社会治安秩序持续稳定。高度重视消防安全，加强道路交通、矿业生产、危爆品等领域的安全监管，加大隐患排查治理力度，深化责任落实，坚决防止重特大事故发生。完善防灾减灾和应急救援体系，提高灾害防御和救助能力。加强国防动员和后备力量建设，深入开展双拥模范城创建活动，推动军民深度融合发展。

（八）强化自身建设，提升政府公信力和执行力

“打铁还得自身硬”。各级政府特别是领导干部，要按照习近平总书记的要求，始终做到“心中有党、心中有民、心中有责、心中有戒”，以政府工作新状态引领经济发展新常态。

推进依法行政。深入贯彻落实党的十八届四中全会精神，切实把政府工作全面纳入法治轨道。认真执行人大及其常委会的决议、决定，依法向人大及其常委会报告工作，自觉接受政协民主监督，认真办理人大代表建议和政协委员提案，积极组织实施市政府和市政协年度协商计划，广泛听取各民主党派、工商联、无党派人士和人民团体的意见建议。严格遵守重大行政决策法定程序，推行政府法律顾问制度。继续深化行政执法体制改革，严格规范公正文明执法。大力推行政务公开，保障人民群众知情权、参与权、表达权和监督权。

强化工作落实。“一打纲领不如一个行动”。我们要牢牢抓住基础性、关键性和长期性的工作，集中精力抓要事、攻难事、成大事，以踏石留印、抓铁有痕的劲头，一抓到底、善作善成，做到有部署、有检查、有问责。干部有多大担当才能干多大事业，尽多大责任才会有多大成就。各级干部不能只想当官不想干事，只想揽权不想担责，只想出彩不想出力，要真正把“为官一任、造福一方”的责任扛在肩上。

建设诚信政府。国以诚立心，人以诚立身。信任是政府存在的基石，打造诚信社会首先要打造诚信政府。各级政府和广大干部要做诚信的倡导者、实践者和维护者，把政府履约和守诺服务作为我们干事创业的根本，树立“功成不必在我”的境界，不论政府换届还是岗位调整，都要保证政策连续性，做到“新官也要理旧账”。

建设廉洁政府。严格落实从严治党八项要求，严守党的政治纪律和政治规矩，坚定自觉地同党中央保持高度一致，坚决贯彻中央、自治区和市委的决策部署。巩固和拓展党的群众路线教育实践活动成果，切实解决好关系群众切身利益的热点、难点问题，推进作风建设常态化。推行权力清单制度，依法公开权力运行流程，加强对职能部门履职情况的监督，坚决整治行政不作为、乱作为。强化对重点领域、关键岗位的行政监察和审计监督。认真落实党风廉政建设主体责任，健全政府系统廉政风险防控体系，让政府的公共权力在阳光下运行。

为政清廉才能取信于民，秉公用权方能赢得人心。我们要把政府自身建设作为抓好一切工作落实的重要保障，全体政府工作人员要严以修身、严以用权、严以律己，谋事要实、创业要实、做人要实，大力提升政府的公信力和执行力，努力建设为民、务实、清廉的政府。

各位代表！事业凝聚人心，实干赢得未来。让我们在市委的坚强领导下，紧紧团结和依靠全市各族人民，脚踏实地，真抓实干，为把通辽建设成为经济发展、社会和谐、各族人民幸福生活的亮丽风景线而努力奋斗！
